# Arthroscopic labral repair concomitantly performed with curved periacetabular osteotomy

**DOI:** 10.1007/s00167-013-2362-x

**Published:** 2013-01-23

**Authors:** Hiroshi Nakayama, Shigeo Fukunishi, Tomokazu Fukui, Shinichi Yoshiya

**Affiliations:** Department of Orthopaedic Surgery, Hyogo College of Medicine, 1-1 Mukogawa-cho, Nishinomiya City, Hyogo 663-8501 Japan

**Keywords:** Hip, Arthroscopy, Osteotomy

## Abstract

A 23-year-old female presented with pain in the left hip. Radiological examination showed developmental dysplasia of the hip (DDH) combined with acetabular retroversion and posterior wall deficiency. Findings in the physical examination were coincident with femoroacetabular impingement. At surgery, we performed curved periacetabular osteotomy concomitant with arthroscopic labral repair and osteochondroplasty, simultaneously addressing dysplastic acetabulum and femoroacetabular impingement. The final follow-up examination at 18 months showed satisfactory outcome with the D’Aubigne and Postel hip score of 17/18. In addition to accurate diagnosis, the arthroscopic procedure for associated intra- and peri-articular problems seems to help improve the surgical outcome of periacetabular osteotomy performed for patients with DDH.

*Level of evidence* IV.

## Introduction

In patients with developmental dysplasia of the hip (DDH), increase in articular stress due to the reduction in contact area leads to gradual osteoarthritic progression with age. In order to prevent this vicious sequel, various forms of periacetabular osteotomy have been reported in the literature. Ganz et al. [4] proposed the Bernese periacetabular osteotomy in 1998. In 2005, Naito et al. [15] reported ‘curved periacetabular osteotomy (CPO)’ as a modification of the Bernese osteotomy. Based on the theoretical advantage of CPO over other osteotomies and the favourable outcomes reported in the literature such as low complication rate [15], the authors have adopted this procedure as a primary surgical option for patients with DDH.

As another factor leading to secondary hip osteoarthritis, the significance of acetabular retroversion associated with femoroacetabular impingement (FAI) has been addressed in recent years [2, 9, 19, 22]. Clinically, acetabular retroversion leads to limited hip flexion and internal rotation caused by impingement of the anterosuperior aspect of the femoral head–neck junction against the acetabular rim. Repetitive stress applied to the anterior edge of the acetabulum by the impingement gives rise to tear and subsequent degenerative changes of the labrum. The resultant labral injuries further add to the sequential process of osteoarthritic progression [5, 9]. With the recent progress in hip arthroscopy and development of arthroscopic management of labral tear, an increasing number of studies dealing with this subject have been reported in the literature [11, 17, 18, 20].

The aforementioned two factors for secondary osteoarthritis (DDH and FAI) can be simultaneously identified in one hip. Moreover, it has been shown that the incidence of FAI is higher in patients with DDH than in the normal population [1–3]. In patients with combined DDH and FAI, either osteotomy or arthroscopic labral procedure alone has been reported to result in a less optimal outcome [6, 13, 16]. With the intent of addressing both problems, the Hyogo College of Medicine current treatment option for patients complicated with these combined pathologies is an arthroscopic labral repair concomitant with CPO. In this preliminary report, the surgical procedure, intraoperative findings and the early outcome of a patient who underwent CPO concomitant with arthroscopic labral repair are presented. To date, surgical intervention with this combined procedure for a patient with DDH has not been reported in previous studies.

## Case report

**Fig. 1:**
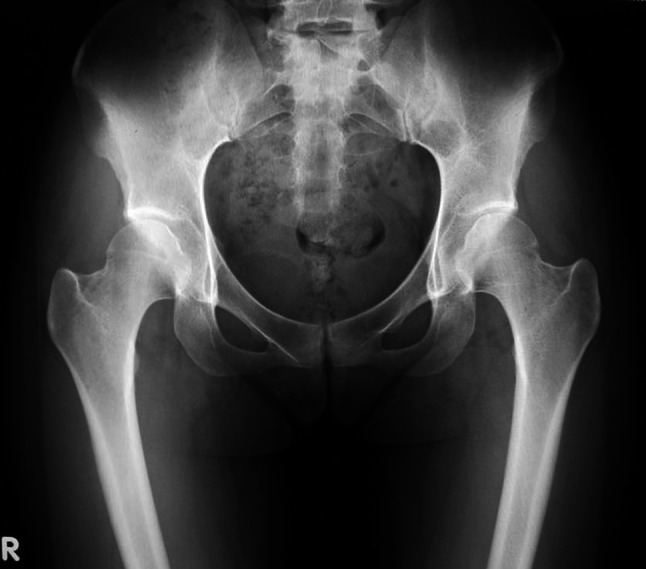
The preoperative anterior–posterior radiograph demonstrated crossover sign at the left acetabulum indicating acetabular retroversion

A 23-year-old female presented with a 1-year history of pain in the left hip. The pain was located in the groin region and her symptom had gradually increased. At her initial visit, a physical examination of the hip revealed limited range of motion, particularly in flexion and internal rotation. Additionally, the anterior impingement test was positive with pain induced on flexion combined with internal rotation. The routine radiological examination (anterior–posterior radiograph) showed findings of DDH with a Sharp angle and centre-edge (CE) angle of 50° and 7°, respectively. The crossover sign and posterior wall sign were identified by radiography and computed tomography indicating the presence of acetabular retroversion (Fig. 1). Based on the clinical and radiological findings, a diagnosis of DDH combined with acetabular retroversion was made for this patient. Considering the persistent nature of pain and disability for the preceding year, surgical intervention was indicated.

**Fig. 2:**
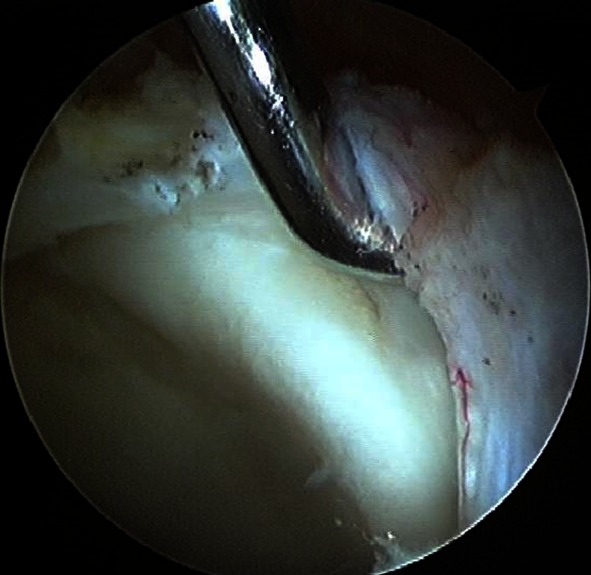
A labrum tear was observed at the anterosuperior edge of the acetabulum. The articular cartilage appeared to be intact

**Fig. 3:**
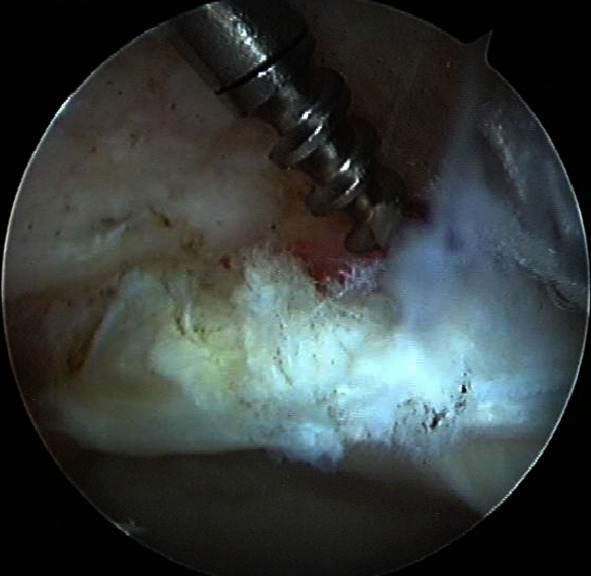
The labrum was fixed back to the acetabulum using two suture anchors. After the labrum repair, osteochondroplasty of the femoral head was performed to prevent anterior impingement

**Fig. 4:**
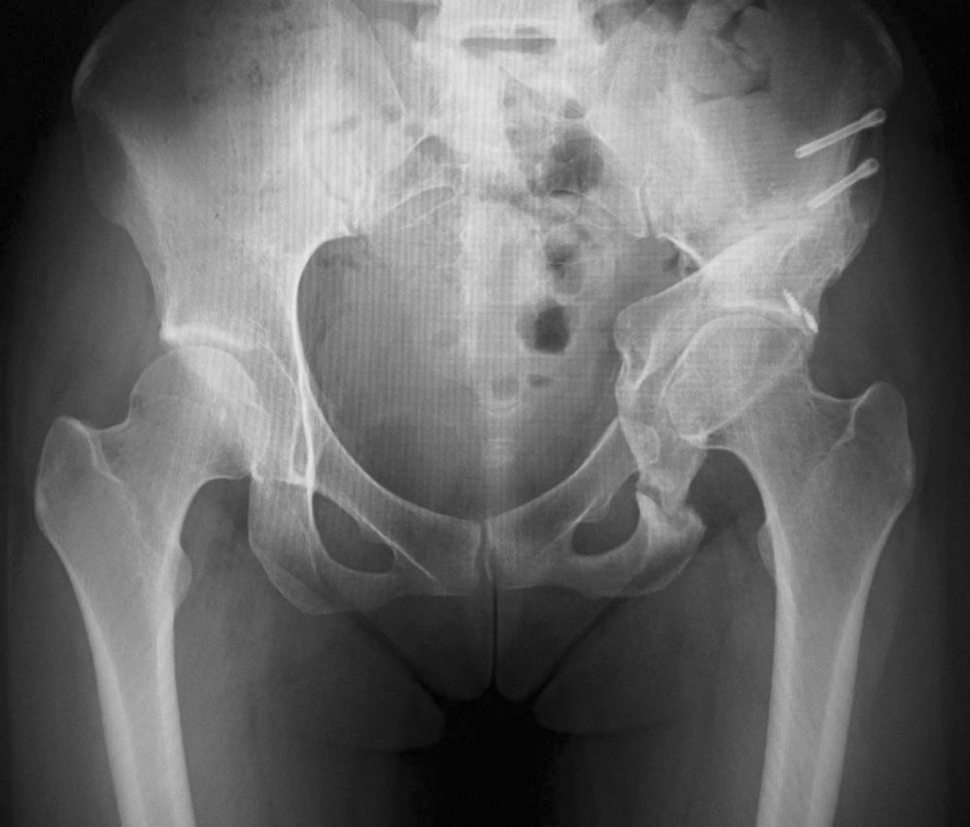
The post-operative anterior–posterior radiograph showed improved acetabular coverage with disappearance of the crossover sign

At surgery, hip arthroscopy was performed prior to CPO. The surgical set-up for hip arthroscopy followed the technique reported by Philippon et al. [18]. Under general anaesthesia, the patient was positioned supine on the fracture table that allowed independent lower extremity traction. Two portals (lateral and anterolateral) were established for the scope and the instruments. The arthroscopic examination revealed a full thickness labral tear at the anterior–superior region (Fig. 2). A labral tear was identified at the anterosuperior edge of the acetabulum, and the tear was fixed back to the rim using two suture anchors (Fig. 3). Additionally, a motorized burr and shaver were used to remove the osteochondral surface and synovium at and around the head–neck junction to avoid impingement on the repaired site. Subsequently, CPO was carried out following the technique described by Naito et al. [15] with the patient repositioned on the operative table in supine position. Three poly-l-lactic acid screws were used to fix the reoriented acetabular fragment, while two double-threaded metal screws were used for the fixation of the osteotomized anterior superior iliac supine. The post-operative radiograph showed improved acetabular coverage of the femoral head with a resultant Sharp angle of 36° and CE angle of 50°, while the crossover sign as well as the posterior wall sign was no longer evident (Fig. 4). Post-operatively, passive motion of the operative hip was initiated on the first post-operative day. Weight-bearing was not allowed for 2 weeks. Thereafter, partial weight-bearing began with progression to full weight-bearing at 8 weeks.

Healing of the osteotomy site and functional recovery were accomplished uneventfully during the subsequent period. Six months after the surgery, the patient returned to her original job as a nurse without left hip pain. The D’Aubigne and Postel hip score improved from 10/18 preoperatively to 17/18 at the 18-month follow-up.

The Review Board of the Hyogo College of Medicine approved this study (No. 1215), and the appropriate written informed consent was obtained from the patient.

## Discussion

The most important finding of the present report was the clinical efficacy of the concomitant arthroscopic labral repair with CPO. Although this surgical approach has not been reported in previous studies, the theoretical advantage of the combined arthroscopic and open procedures seems clear considering the mechanism of the osteoarthritic progression.

FAI has been proposed as a condition that gives rise to excessive contact stress between the acetabular rim and the femoral head–neck junction potentially leading to osteoarthritis [5, 12]. Repetitive impingement occurring at the anterior rim of the acetabulum causes tear and degeneration of the labrum, and retroversion of the acetabulum is thought to be the principal anatomic characteristic observed in this type of impingement [9, 19].

The prevalence of acetabular retroversion in the normal population as well as patients with hip disorders has been investigated [1–3]. Fujii et al. [3] reported acetabular retroversion in 17 of the 96 hips (18 %) of patients with DDH. Also, Ezoe et al. [1] reported the prevalence in 18 % (13 of 74 hips) of DDH patients as compared to 6 % (7 of 112 hips) in normal subjects. These reported results show that the incidence of acetabular retroversion is higher in the DDH population. The patient in this case report exhibited radiographic features indicating deficiency of the lateral coverage of the femoral head. In addition, crossover as well as posterior wall sign was evident indicating an accompanied acetabular retroversion.

Regarding the surgical treatment for DDH, various forms of periacetabular osteotomy have been proposed and reported with their surgical outcome [7, 15]. Although these procedures can effectively improve lateral coverage of the femoral head, inadvertent post-operative acetabular retroversion has also been reported [10, 14, 21]. Myers et al. [14] reported five cases presenting anterior FAI caused by acetabular retroversion following the Bernese periacetabular osteotomy. Xie et al. [21] evaluated the acetabular version after CPO and showed that post-operative retroversion was identified in 62 % of their patient population, while Kiyama et al. [10] reported that 5 of 24 hips (24 %) with acetabular retroversion following CPO were complicated with post-operative progression of osteoarthritis. These reports indicate the significance of addressing FAI in the planning and performing of periacetabular osteotomy for patients with DDH.

Hip arthroscopy offers a minimally invasive technique for diagnosis as well as therapeutic management of FAI [11, 17, 18, 20]. In regard to the arthroscopic management, it has been reported that an arthroscopic labral procedure alone could not benefit the patients with the evidence of abnormal hip morphologies [6, 13, 16]. Kim et al. [8] reported that labral lesions were detected at arthroscopy in 88.4 % of the patients who underwent periacetabular rotational osteotomy. They performed arthroscopic shaving or debridement for those lesions and concluded that the concomitant arthroscopic treatment for intra-articular pathology during periacetabular osteotomy may improve the functional and radiological outcome. In their report, however, labrum repair was not attempted. Recently, there have been some comparative clinical studies showing that labral repair provides superior results as compared to labral resection or debridement [11, 17, 20]. However, there have been no published reports of arthroscopic labral repair concomitantly performed with periacetabular rotational osteotomy in patients with DDH. This is the first report of arthroscopic labral repair in conjunction with periacetabular rotational osteotomy for a patient with DDH, achieving a satisfactory short-term outcome. However, a long-term follow-up study including a large number of patients would be required to critically assess the efficacy of our treatment approach.

## Conclusion

Arthroscopic labral repair concomitant with CPO was performed in a patient with DDH and an associated labral tear. The post-operative course was uneventful and the patient returned to her original job as a nurse 6 months after surgery without pain. Labral tear secondary to FAI combined with acetabular retroversion is known to be a risk factor for osteoarthritic progression, while the incidence of retroversion is higher in patients with DDH. Therefore, arthroscopic management for FAI in conjunction with periacetabular osteotomy for this patient population as reported in this article is theoretically advantageous for maximizing the surgical benefit.

## References

[1] Ezoe M, Naito M, Inoue T (2006). The prevalence of acetabular retroversion among various disorders of the hip. J Bone Joint Surg Am.

[2] Fujii M, Nakashima Y, Sato T, Akiyama M, Iwamoto Y (2011). Pelvic deformity influences acetabular version and coverage in hip dysplasia. Clin Orthop Relat Res.

[3] Fujii M, Nakashima Y, Yamamoto T, Mawatari T, Motomura G, Matsushita A, Matsuda S, Jingushi S, Iwamoto Y (2010). Acetabular retroversion in developmental dysplasia of the hip. J Bone Joint Surg Am.

[4] Ganz R, Klaue K, Vinh TS, Mast JW (1988). A new periacetabular osteotomy for the treatment of hip dysplasia. Technique and preliminary results. Clin Orthop Relat Res.

[5] Ganz R, Parvizi J, Beck M, Leunig M, Nötzli H, Siebenrock KA (2003). Femoroacetabular impingement: a cause for osteoarthritis of the hip. Clin Orthop Relat Res.

[6] Kain MS, Novais EN, Vallim C, Millis MB, Kim YJ (2011). Periacetabular osteotomy after failed hip arthroscopy for labral tears in patients with acetabular dysplasia. J Bone Joint Surg Am.

[7] Karashima H, Naito M, Shiramizu K, Kiyama T, Maeyama A (2011). A periacetabular osteotomy for the treatment of severe dysplastic hips. Clin Orthop Relat Res.

[8] Kim KI, Cho YJ, Ramteke AA, Yoo MC (2011). Peri-acetabular rotational osteotomy with concomitant hip arthroscopy for treatment of hip dysplasia. J Bone Joint Surg Br.

[9] Kim WY, Hutchinson CE, Andrew JG, Allen PD (2006). The relationship between acetabular retroversion and osteoarthritis of the hip. J Bone Joint Surg Br.

[10] Kiyama T, Naito M, Shiramizu K, Shinoda T (2009). Postoperative acetabular retroversion causes posterior osteoarthritis of the hip. Int Orthop.

[11] Larson CM, Giveans MR (2009). Arthroscopic debridement versus refixation of the acetabular labrum associated with femoroacetabular impingement. Arthroscopy.

[12] Leuning M, Beaulë E, Ganz R (2009). The concept of femoroacetabular impingement. Current status and future perspectives. Clin Orthop Relat Res.

[13] Mei-Dan O, McConkey MO, Brick M (2012). Catastrophic failure of hip arthroscopy due to iatrogenic instability: can partial division of the ligamentum teres and iliofemoral ligament cause subluxation?. Arthroscopy.

[14] Myers SR, Eijer H, Ganz R (1999). Anterior femoroacetabular impingement after periacetabular osteotomy. Clin Orthop Relat Res.

[15] Naito M, Shiramizu K, Akiyoshi Y, Ezoe M, Nakamura Y (2005). Curved periacetabular osteotomy for treatment of dysplastic hip. Clin Orthop Relat Res.

[16] Parvizi J, Bican O, Bender B, Mortazavi SM, Purtill JJ, Erickson J, Peters C (2009). Arthroscopy for labral tears in patients with developmental dysplasia of the hip: a cautionary note. J Arthroplasty.

[17] Philippon MJ, Schroder e Souza BG, Briggs KK (2010). Labrum: resection, repair and reconstruction sports medicine and arthroscopy review. Sports Med Arthrosc.

[18] Philippon MJ, Stubbs AJ, Schenker ML, Maxwell RB, Ganz R, Leunig M (2007). Arthroscopic management of femoroacetabular impingement: osteoplasty technique and literature review. Am J Sports Med.

[19] Reynolds D, Lucas J, Klaue K (1999). Retroversion of the acetabulum. A cause of hip pain. J Bone Joint Surg Br.

[20] Schilders E, Dimitrakopoulou A, Bismil Q, Marchant P, Cooke C (2011). Arthroscopic treatment of labral tears in femoroacetabular impingement: a comparative study of refixation and resection with a minimum two-year follow-up. J Bone Joint Surg Br.

[21] Xie J, Naito M, Maeyama A (2010). Evaluation of acetabular versions after a curved periacetabular osteotomy for dysplastic hip. Int Orthop.

[22] Yasunaga Y, Yamasaki T, Matsuo T, Ishikawa M, Adachi N, Ochi M (2010). Crossover sign after rotational acetabular osteotomy for dysplasia of the hip. J Orthop Sci.

